# Systematic review and meta-analysis of clinical outcomes comparison between different initial dialysis modalities in end-stage renal disease patients due to lupus nephritis prior to renal transplantation

**DOI:** 10.1186/s12882-020-01811-y

**Published:** 2020-05-01

**Authors:** Joel Swai, Xiexiong Zhao, Julie-Raisa Noube, Gui Ming

**Affiliations:** 1 Department of Nephrology and Rheumatology, Xiangya Third Hospital, Central South University, Changsha City, Hunan Province People’s Republic of China; 2 Department of Nephrology, Benjamin Mkapa Hospital, Dodoma City, Dodoma Region United Republic of Tanzania; 3 Department of Cardiology, Xiangya Third Hospital, Central South University, Changsha City, Hunan Province People’s Republic of China; 4 Department of Gastroenterology, Xiangya Third Hospital, Central South University, Changsha City, Hunan Province People’s Republic of China

**Keywords:** Renal Dialysis, Lupus nephritis, Kidney failure, chronic, Meta-analysis

## Abstract

**Background:**

Regarding lupus disease activity, morbidity and survival, limited literature concluded conflicting results when comparing hemodialysis versus peritoneal dialysis as initial renal replacement therapies (RRT) prior to transplantation, in lupus nephritis end-stage renal disease (LN-ESRD) patients. This study was aimed to compare the risks of lupus flares, all-cause infections, all-cause cardiovascular events, and mortality, between hemodialysis versus peritoneal dialysis as initial RRT - modality before renal-transplant in LN-ESRD patients, by systematic review and meta-analysis.

**Methods:**

PubMed, EMBASE, and SCOPUS were searched for observational-studies comparing LN-ESRD -patients undergoing hemodialysis (Group1) versus peritoneal-dialysis (Group 2) prior to renal-transplantation, by their risks of lupus flare, all-cause infections, all-cause cardiovascular events, and mortality as outcome measures. Relative-Risks of outcomes between the groups measured overall effects at a 95% significance level. RevMan 5.3 computer software was used for analysis.

**Results:**

From search, 16 eligible studies reported 15,636 LN-ESRD -patients prior to renal transplantation with 4616 patients on hemodialysis, 2089 on peritoneal dialysis, 280 directly underwent kidney transplantation, 8319 were eliminated with reasons and 332 participants’ details were not reported. Hemodialysis group had higher risk of all-cause cardiovascular events, Relative-Risk = 1.44 (Confidence Interval:1.02, 2.04), *p*-Value< 0.05. With regards to risks for mortality, flare and all-cause infections, there were trends that were not statistically significant (p-Value> 0.05).

**Conclusion:**

Except for all-cause cardiovascular events in which peritoneal dialysis is superior to hemodialysis offering better outcomes, both treatment modalities offer more or less similar clinical outcomes as effective initial choices of RRT in LN-ESRD patients prior to renal transplant.

**The protocol registration:**

PROSPERO 2019 CRD42019131600.

## Background

End-stage renal disease (ESRD) is an advanced stage of progressively function-loss of kidneys, commonly characterized by an estimated glomerular filtration rate (eGFR) of lower than 15 ml per minute per 1.73 square meters. ESRD results from an ultimate complication of underlying renal debilitating chronic conditions that could range from systemic diseases such as diabetes, hypertension, inflammatory conditions such as glomerulonephritis and tubulointerstitial nephritis, autoimmune disorders like systemic lupus erythematosus (SLE), genetic disorders including polycystic kidney diseases to chronic urinary tract infections and obstructive conditions [1–4].

Being a systemic disease, SLE manifestations spin from causing mucocutaneous inflammations, neurological symptoms, arthritis, and pancytopenia to multi-organ failures. SLE pathogenesis results from the formation of autoantibodies, activation of serum complements, and deposition of immune complexes in various tissues followed by the initiation of inflammation. The deposition of these immune complexes and their associated inflammations in the kidneys result in lupus nephritis [5]. About 60% of SLE patients will eventually complicate to lupus nephritis, with male gender and black ancestry in preference [1, 6].

About 10% of all lupus nephritis patients will eventually progress to ESRD which may necessitate RRT such as hemodialysis, peritoneal dialysis and/or kidney transplant [7]. Previous literature has shown that the choice of RRT modality strongly depends on ethnicity [8], employment status, medical insurance type [8, 9] and comorbidities burden [10]. Behind diabetes mellitus and hypertension which are by far the major causes of ESRD in adults, by the year 2012, lupus nephritis constituted 1.60% of all ESRD patients in the United States of America [11]. In children, however, during the 2009–2013 period, cystic and congenital disorders constituted the leading causes of ESRD with 33% ahead of glomerular disease (24.60%), and other secondary causes of glomerulonephritis which constituted about 13% [12].

Hemodialysis and peritoneal dialysis serve as initial RRT modalities prior to kidney transplantation which is considered a superior modality of the 3, in terms of patients’ survival and quality of life [13, 14]. On adequate immunosuppressive drugs, 10 to 28% of LN-ESRD patients on dialysis will improve enough not to require dialysis any further [15]. Therefore, though debatable, a short period of time (i.e. not exceeding 24 months [16]) on dialysis after developing ESRD due to lupus nephritis is advised, before transplantation is opted [15, 17, 18].

In terms of SLE disease activity and morbidity, a few publications available have concluded conflicting results when comparing hemodialysis versus peritoneal dialysis as initial RRT modalities prior to renal transplantation in LN-ESRD patients. *Tsai* [19] opposed by *Krane* [20], reported more SLE disease activity in hemodialysis patients than in peritoneal dialysis, Chang [21] opposed by *Kang* [13], reported higher risk of infections in hemodialysis than peritoneal dialysis patients, *Tsai* [19] opposed by *Weng* [22], reported a higher risk of all-cause cardiovascular events in hemodialysis than peritoneal dialysis and Wu [23] opposed by *Contreras* [24], reported a higher risk of mortality in hemodialysis than in peritoneal group. *Ntatsaki* et al. [16] in a large study reported similar risks of mortality between the groups.

Therefore, this study will compare between hemodialysis versus peritoneal dialysis modalities in terms of the risks for disease activity, all-cause infection, all-cause cardiovascular events, and mortality in LN-ESRD adult patients, as initial RRT modality before renal transplant, by systematic review and meta-analysis of available literature.

## Methods

### Study registration

The protocol for this study was registered at PROSPERO 2019 CRD42019131600 and it can be found via the following link; https://www.crd.york.ac.uk/prospero/display_record.php?RecordID=131600

### Eligibility criteria

This study included participants with ESRD (i.e. eGFR of lower than 15 ml per minute per 1.73 square meters) due to lupus nephritis receiving either of the 2 initial RRT namely, hemodialysis or peritoneal dialysis, prior to renal transplant. Both adults (i.e. more or equal to 18 years of age) and pediatric (i.e. less than 18 years old) participants were eligible for inclusion. The main outcomes were; risks of lupus flare, all-cause infections, all-cause cardiovascular events and mortality. Both prospective and retrospective conducted matched case-control studies comparing the suitable outcomes between the 2 initial dialysis modalities in LN-ESRD were eligible for inclusion. To increase the external validity of this study, accessible literature from all around the world were eligible for inclusion. Only English publications were eligible for inclusion.

### Information sources

The 3 online databases, namely PubMed, EMBASE and the SCOPUS were searched to come up with eligible included studies. The searches were not customized for searching within any restricted date ranges. Secondary referencing of eligible studies was done to extend the search scope. The last date of the search was 28th September 2019.

### The search

To generate a set of citations that were relevant to our study’s search question, an advanced search tool was used in all of the 3 databases aforementioned. Using PubMed, MeSH search builder was utilized; ((“Kidney Failure, Chronic”[MeSH] AND “Renal Replacement Therapy”[MeSH]) AND “Renal Dialysis”[MeSH]) AND “Lupus Nephritis”[MeSH] AND “humans”[MeSH Terms]. The search was Repeated with; (((“Lupus Nephritis”[MeSH] AND “Peritoneal Dialysis”[MeSH]) AND “Renal Dialysis”[MeSH]) AND “Kidney Transplantation”[MeSH]) AND “Kidney Failure, Chronic”[MeSH] AND “humans”[MeSH Terms]. Furthermore, a combination of keywords (non-Mesh) was also used to provide more results. These searches were independently performed by 2 authors; JS and XZ. Results were exported to computer software, *EndNote X9 (Bld 12,062)* which was used to manage and keep track of references throughout this study.

### Study selection process

**Fig. 1:**
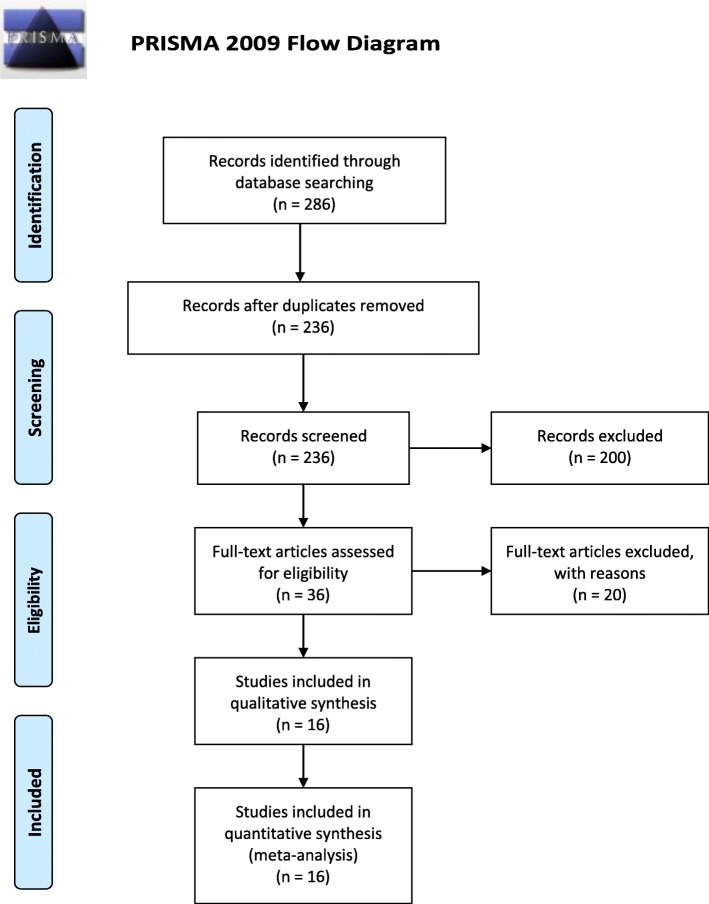
PRISMA 2009 Flow Diagram for study selection

All studies resulting from the online database search, independently conducted by 2 authors, were screened by their titles and abstracts to initially assess their relevance to our study question. This was, the first-level screening, and was done by the same 2 authors; JS and XZ. Compiled results of first-level screening were then searched for their full-text articles. Second-level scrutiny involved assessing the retrieved full-text articles for eligibility for inclusion or exclusion. Any differences of thoughts in the search process were settled by the third author, JN. The search process is summarized in Fig. 1.

### Data extraction

Before data was extracted from full-text articles meeting eligibility criteria for inclusion, assessment for methodological biases was done using the Newcastle - Ottawa quality assessment scale [25]. PRISMA (preferred reporting items for systematic reviews and meta-analyses) tool [26] was used for this study write-up to minimize reporting bias.

The process of data extraction was independently performed by 2 authors, namely JS and XZ. Any difference in thoughts was settled by the third author, GM. Data collected included participants’ demographics, study characteristics and reported clinical outcomes in line with our study question.

Demographic data included participants’ mean age and ethnicity. Modality of dialysis used, whether hemodialysis or peritoneal dialysis and the number of participants undertaking the modality prior to renal transplant were also recorded. Follow-up time and whether a participant switched to another treatment modality during the follow-up period was recorded as well. These participants were later eliminated from the analysis.

In line with this study question, outcomes recorded from the eligible studies included; risks of lupus flare, all-cause infection, all-cause cardiovascular events, and mortality. These outcomes were recorded depending on the treatment group of occurrences i.e. hemodialysis group or peritoneal dialysis group. The risk was defined as the number of participants developing an outcome of interest (i.e. all-cause infection, flare, all-cause cardiovascular event or mortality) during the study follow-up period, divided by total number of participants in the treatment group.

### Analysis

Data were analyzed separately according to the outcomes of interest. This gave rise to 4 separate analyses; comparison of risk of all-cause infections between hemodialysis and peritoneal dialysis groups; comparison of risk of lupus flares between hemodialysis and peritoneal dialysis groups; comparison of risk of all-cause cardiovascular events between hemodialysis and peritoneal dialysis groups; and lastly comparison of risk of mortality between hemodialysis and peritoneal dialysis groups. Risk ratio (RR) was used to measure and compare outcomes modified by the 2 dialysis modalities.

The overall effects of dialysis modalities were diagrammatically depicted by forest-plots. Data synthesis, analysis, and generation of forest-plots were done utilizing computer software, ***Review Manager (RevMan Version 5.3)***. The software was customized to a random or fixed effect model depending on the heterogeneity (I^2^) of the studies when analyzing the outcomes. The fixed-effect model was used when I^2^ was less than 50% and the random effect model was used if I^2^ was more than 50%.

### Assumptions and simplifications

For this study purpose, all participants were considered to have been correctly diagnosed with end-stage renal disease strictly due to lupus nephritis and not due to other causes of ESRD such as diabetes or hypertension. Amid 6 guidelines to manage lupus nephritis [27], none is currently specified for LN-ESRD, hence authors assumed that all participants, despite study country had received standard care aligning with internationally accepted guidelines with KDIGO (Kidney Disease- Improving global outcomes – CKD evaluation and management) [28].

## Results

### Search

Preliminary search from online databases using a combination of terms in the advanced search tool and MeSH terms resulted in 302 studies. Of 302 studies, 33 were duplicates, hence discarded. Of 269 remaining studies, 84 were from PubMed, 102 from EMBASE and 83 were from SCOPUS. These were exported to EndNote.

First level scrutiny i.e. screening titles and abstracts, resulted in the elimination of 233 studies as were irrelevant to our study question. Full-text articles for the remaining 66 studies were sought and were screened for inclusion and exclusion criteria i.e. Second level scrutiny. Full-text articles of 3 otherwise eligible studies, [7], [17] and [29] were published in Italian, German and Croatian respectively, hence excluded. A study by *Goo* et al. [30], was excluded because it did not report the exact number of patients developed our outcome of interests, only reported increase in maximum SLE Disease Activity Index (SLEDAI) score after RRT and insignificant difference at 2, 5 and 10 year survival between the modalities. After the second scrutiny and elimination of duplicate studies, 16 studies were ultimately eligible for inclusion in this review and meta-analysis.

### Study characteristics

A characteristic summary of 16 articles included in this study is illustrated in Table 1**.** From 16 ultimately eligible studies, 15,636 patients were diagnosed to have LN-ESRD prior to renal transplantation. Of 15,636 patients, 4616 were on hemodialysis and 2089 were on peritoneal dialysis, 280 directly underwent kidney transplantation, 8319 were eliminated in 1 study [24] after matching participants utilizing propensity scores by the primary author and for 332 participants details were not reported [16].

**Table 1:** Study characteristics

Study	Participants Number	Dialysis modality HD, PD	Median Age HD, PD	Ethnicity/Race	Follow-up time HD; PD	Primary study aim	Country of study	Outcome measure
Kang 2011 [13]	59	28, 14	35, 41	Not Reported	5 ± 3; 5 ± 3 (Years)	Long-term outcome of patients with ESRD secondary to SLE who are managed by different types of RRTs	South Korea	**LFR, IR, MR, PS**
Tsai 2019 [19]	94	42, 12	36.40, 33.20	Not Reported	6.30 ± 5.10; 6.00 ± 5.20 (Years)	Long-term outcomes and survival rates of patients with ESRD caused by lupus nephritis who received 3 different modalities of renal replacement therapy	Taiwan	**LFR, IR, MR, CVR, PS**
Krane 1999 [20]	19	7, 5	32, 36	10 black, 2 Whites	3 years; 3 years	Lupus activity among patients with ESRD due to SLE, who were either undergoing dialysis or had undergone transplantation	USA	**LFR**
Chang 2013 [21]	1073	813, 260	42.60, 34.10	Not reported	≥ 3 months on RRT; ≥ 3 months on RRT	Mortality and the impact of dialysis modalities on the survival in SLE patients with end-stage renal disease (ESRD).	Taiwan	**IR, MR, CVR, PS**
Weng 2009 [22]	36	14, 22	48.70, 37.59	Not reported	126.83; 37 (Months)	Comparing PD and HD outcomes between female SLE patients with ESRD due to lupus nephropathy	Taiwan	**IR, CVR, MR, PS**
Wu 2014 [23]	1998	1640, 196	39.30, 36.20	Not Reported	3.31 ± 3.87; 4.34 ± 3.05 (Years)	Outcomes of patients with LN after progression to ESRD and to try to elucidate whether deferral of KT is necessary in the Chinese population.	Taiwan	**MR, PS**
Contreras 2014 [24]	11,023	1352, 1352	39,39	Caucasian American; African American; Asian Americans; Other Americans.	3 years; 3 years (Median)	Comparing the mortality risk of ESRD patients with SLE initiating with PD versus HD	USA	**MR**
Stock 1993 [31]	6	6, 6	Not Accessed	Not Accessed	Not Accessed	Determining if there was a difference in disease activity between treatment modalities, using patients as self-controls	USA	**LFR**
Zhu 2009 [32]	29	10, 19	34.50, 41.79	Not Reported	2 years; 2 years	Comparing 2-year outcome of ESRD in lupus nephritis patients in different dialysis modality.	China	**LFR, IR, MR, CVR, PS**
Ntatsaki 2018 [16]	361	17, 9	Not Accessed	Caucasian; Afro–Caribbean; South Asian	43 (Confidence interval13–49) (Months)	Investigating the time spent on dialysis before RT and survival ollowing RT in a cohort of SLE patients.	UK	**MR**
Zhang 2016 [33]	425	314, 111	Not Accessed	European; Maori and Pacific Islanders; Asian; Others	3.80 Years (Median); 3.80 Years (Median)	Comparing dialysis and transplant outcomes for patients with ESRD due to lupus nephritis to all other causes	Australia	**MR**
Levy 2015 [34]	368	308, 60	43.50, 43.90	Not Reported	5 Years; 5 Years	Describing the outcomes of SLE on chronic dialysis	France	**MR, CVR**
Mustapic 2013 [35]	7	6, 1	Not Accessed	Not Reported	Up to 10 years	Evaluating outcomes of pediatric patients with ESRD due to lupus nephritis and to determine has intensive specific treatment in SLE decreased incidence of ESRD and need for RRT, dialysis and kidney transplantation, in pediatric patients in the last 4 decades in Croatia	Croatia	**MR**
Kang 2010 [36]	59	28, 14	Not Accessed		5 ± 3 Years; 5 ± 3 Years;	Demonstrating the long-term outcome of lupus patients that underwent different RRTs including kidney transplantation	South Korea	**LFR, CVR**
Oliveira 2012 [37]	50	11, 2	Not Accessed	Non-Caucasians	11 Months;30 Months	Determining the epidemiological profile and outcome of patients with LN undergoing renal transplantation.	Brazil	**LFR, MR**
Lee 2003 [38]	26	20, 6	Not Accessed	Not recorded	57.50 ± 4.20 Months	Investigating the long-term prognosis of 26 SLE patients who started regular dialysis at a Chinese hospital whose stay exceeded a 3-month duration.	Taiwan	**MR**

*Abbreviations*: *IR* infection risk, *LFR* lupus flare risk, *CVR* cardiovascular events risk, *MR* mortality risk, *PS* patients survival, *LN* lupus nephritis, *RRT* renal replacement therapy, *SLE* systemic lupus erythematosus, *RT* renal transplant, *PD* peritoneal dialysis, *HD* hemodialysis

All 16 studies reported 1 or more outcomes of interest. Studies reporting similar outcomes of interest were analyzed together. A total of 7 studies compared lupus flare risk [13, 19, 20, 31, 36, 37] between the 2 dialysis modality groups, 5 compared all-cause infection risk [13, 19, 21, 22, 36], 6 compared all-cause cardiovascular events risk [13, 19, 21, 22, 34, 36] and 12 compared the risk of mortality [13, 16, 19, 21–24, 33–35, 37, 38] between the 2 dialysis modality groups.

A total of 5 studies were conducted in Taiwan [19, 21–23, 38], 3 in the United States of America (USA) [20, 24, 31], 1 in China [32], UK, Australia, France, Croatia, Brazil, and 2 in South Korea [13, 36]. All studies were retrospectively conducted studies while 2 [22, 38] were prospectively conducted.

### Sources of bias

All 16 eligible studies included in this study were assessed for risk of bias using the ***Newcastle - Ottawa quality assessment scale*** (Table 2). Sample sizes for participants differed from study to study. Other studies had larger sample sizes [24] while other studies had as smaller sample sizes [31]. Larger sample sizes are more likely to represent the general population (i.e. Generalizability) than are smaller sample sizes. Furthermore, none of these 16 eligible studies showed to have calculated required sample sizes prior to conducting the studies.

**Table 2:** Study bias by Newcastle - Ottawa quality assessment scale

Study	Selection	Comparability	Exposure
Kang 2011 [13]	★★★	★	★★★★
Tsai 2019 [19]	★★★	★	★★★★
Krane 1999 [20]	★★★	★	★★★★
Chang 2013 [21]	★★★	★	★★★★
Weng 2009 [22]	★★★	★	★★★★
Wu 2014 [23]	★★★	★	★★★★
Contreras 2014 [24]	★★★	★★	★★★★
Stock 1993 [31]	★★★	★	★★★★
Zhu 2009 [32]	★★★	★	★★★★
Ntatsaki 2018 [16]	★★	★	★★★★
Zhang 2016 [33]	★★★	★	★★★★
Levy 2015 [34]	★★★	★	★★★★
Mustapic 2013 [35]	★★★	★★	★★★★
Kang 2010 [36]	★★★	★	★★★★
Oliveira 2012 [37]	★★	★	★★★★
Lee 2003 [38]	★★★	★	★★★★

★ - Score

Except for Contreras [24], who matched the 2 comparison groups by age, 15 other studies used different mean-aged groups to compare outcomes in hemodialysis versus peritoneal dialysis groups. Furthermore, 2 same comparison groups, say hemodialysis group, from 2 different studies, had different mean-age of their participants. This reduces the comparability of the studies and increases heterogeneity.

Both retrospective [13, 21] and prospective study [22] designs were found to be eligible for inclusion in this study. Generally, prospectively conducted studies have fewer chances of bias and confounders as compared to retrospectively conducted studies. Prospective studies have lesser information and recall bias risks for bias than retrospective studies. On the other hand, retrospective studies have lesser attrition bias risks. All studies reported having used American College of Rheumatology criteria to diagnose SLE hence low selection biases. Furthermore, the dialysis modality utilized was obtained from patients’ records hence mitigating selection biases. However, despite a number of factors reported to be influencing the selection of either HD or PD [9, 39], physicians’ clinical judgment could be playing a role as a clinically sicker patient is likely to be administered more aggressive approaches as HD than PD, inducing selection biases.

Sampling participants from various countries could be beneficial in increasing generalizability but on the other hand, could mean different treatment guidelines or access to healthcare hence reduce comparability. Also, of all 16 studies, none is from a country from Africa thus less representative of the world’s population. This creates bias as different ethnicities have also been reported to have different clinical outcomes [11] in regards to SLE outcomes, with Black having the worst course than Caucasians.

Despite the fact that all participants had ESRD due to lupus nephritis, 3 [21, 23, 24] studies reported having had LN-ESRD participants with other comorbidities. Different comorbidities in different participants are sources of biases and reduce comparability. Furthermore, comparing all-cause infection risk between the 2 groups could be confounded by different doses of immunosuppressive drugs among participants as per their SLE disease severity. The higher the burden of immunosuppressive drugs would mean the higher the probability of infections.

### Lupus flare risk

**Fig. 2:**
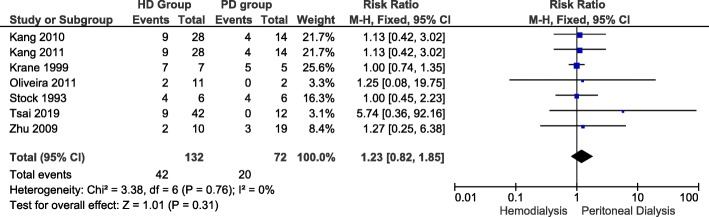
Risk of lupus flares in LN-ESRD undergoing hemodialysis and those undergoing peritoneal dialysis

Figure 2 illustrates 7 of 16 studies that reported risk of lupus flares in LN-ESRD undergoing hemodialysis and those undergoing peritoneal dialysis. In hemodialysis group flares risk ranged from 0.18 [37] to 1 [20]. In peritoneal dialysis group, flare risk ranged from 0 [19] to 1 [20]. The overall risk ratio (RR) of lupus flare between the 2 groups was 1.23 (Confidence Interval: 0.82, 1.85). The difference that hemodialysis is associated with more lupus flares, did not reach statistical significance (*P*-value = 0.31). Fixed-effect model was used since heterogeneity, I^2^, was 0% (i.e. I^2^ < 50%).

### All-cause cardiovascular events risk

**Fig. 3:**
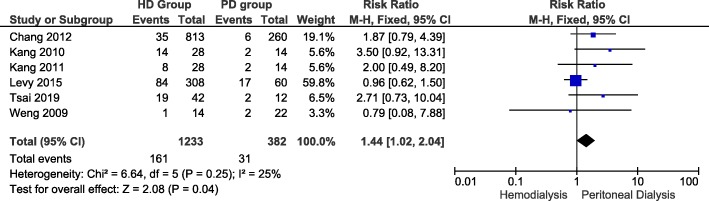
All-cause cardiovascular events in LN-ESRD undergoing hemodialysis and peritoneal dialysis

Figure 3 illustrates 6 of 16 studies that reported risk of all-cause cardiovascular events in LN-ESRD undergoing hemodialysis and peritoneal dialysis. In hemodialysis group, risk of all-cause cardiovascular events ranged from 0.04 [21] to 0.45 [19]. In the peritoneal dialysis group, risk of all-cause cardiovascular events ranged from 0.25 [21] to 0.16 [19]. The overall risk ratio between the groups was 1.44 (Confidence Interval: 1.02, 2.04). The difference that hemodialysis is associated with more all-cause cardiovascular events than peritoneal dialysis, reached statistical significance (*P*-value = 0.04). A fixed-effect model was used since heterogeneity, I^2^, was 25% (i.e. I^2^ < 50%).

### All cause infection risk

**Fig. 4:**
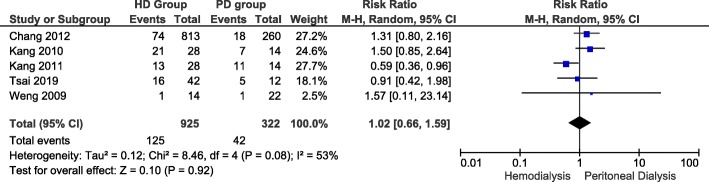
All-cause infection risk in LN-ESRD undergoing hemodialysis and peritoneal dialysis

Figure 4 illustrates 5 of 16 studies that reported risk of all-cause infections in LN-ESRD undergoing hemodialysis and peritoneal dialysis. From hemodialysis group, infection rate ranged from 0.09 [21] to 0.46 [13]. From peritoneal dialysis group, all-cause infection risk ranged from 0.07 [21] to 0.79 [13]. The overall risk ratio between the 2 groups was 1.02 (Confidence Interval: 0.66, 1.59). This difference that peritoneal dialysis is associated with lesser all-cause infection risk, did not reach statistical significance (*P*-value = 0.92). A random-effect model was used since heterogeneity, I^2^, was 53% (i.e. I^2^ > 50%).

### Mortality risk

**Fig. 5:**
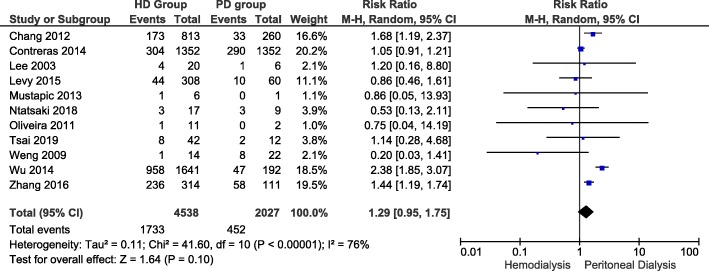
Risk of mortality of LN-ESRD patients undergoing hemodialysis and those undergoing peritoneal dialysis

Figure 5 illustrates 11 of 16 eligible studies that reported risk of mortality of LN-ESRD patients undergoing hemodialysis and those undergoing peritoneal dialysis. In hemodialysis group, the risk ranged from 0.07 [22] to 0.58 [23]. In peritoneal dialysis group, mortality risk ranged from 0.13 [21] to 0.29 [13]. The overall risk ratio between the 2 groups was 1.29 (Confidence Interval: 0.95, 1.75). The difference that hemodialysis is associated with higher risk of mortality than peritoneal dialysis, did not reach statistical significance (P-value = 0.10). A random-effect model was used since heterogeneity, I^2^, was 76% (i.e. I^2^ > 50%).

### Sensitivity analysis

Following a high heterogeneity observed in analyzing risk of mortality, we attempted to eliminate 1 peculiar study, *Mustapic* et al. (2013), whom unlike others, assessed pediatric patients. However, the statistical significance on mortality did not change significantly. The newly obtained overall risk ratio was 1.29 (Confidence Interval: 0.95, 1.76), I^2^ = 78%, *p*-Value = 0.10.

## Discussion

Many studies have compared clinical outcomes between LN-ESRD patients versus ESRD patients due to other causes like diabetes and hypertension. Many have also compared clinical outcomes between different RRT in ESRD due to various causes. Only a few studies have compared clinical outcomes of different RRT in lupus nephritis-caused ESRD. Furthermore, of few studies comparing the RRT in lupus nephritis-caused ESRD, conflicting results about their clinical outcomes have been reported. Our study compared patients’ clinical outcomes between LN-ESRD undergoing hemodialysis versus LN-ESRD undergoing peritoneal dialysis prior to a kidney transplant.

From our study, hemodialysis was associated with higher lupus flare risk than peritoneal dialysis, RR = 1.23 (Confidence Interval: 0.82, 1.85) but the difference did not reach statistical significance (*P*-value = 0.31), *P* > 0.05. Hemodialysis was associated with higher all-cause infection risk than peritoneal dialysis, 1.02 (Confidence Interval: 0.66, 1.59) but the difference did not reach statistical significance, (*P*-value = 0.92), *P* > 0.05. Hemodialysis was associated with higher risk of all-cause cardiovascular events than peritoneal dialysis, 1.44 (Confidence Interval: 1.02, 2.04) and the difference reached statistical significance, (*P*-value = 0.04). *P* < 0.05. Hemodialysis was associated with higher risk of mortality, 1.29 (Confidence Interval: 0.0.95, 1.75) than peritoneal dialysis but the difference did not reach statistical significance, (*P*-value = 0.10), *P* > 0.05. From these results, despite statistical insignificance of all-cause infection risk, lupus flare risk, and mortality, peritoneal dialysis in LN-ESRD is superior to hemodialysis as an initial RRT of choice prior to renal transplant, in terms of better cardiovascular outcomes.

Higher risk of all-cause cardiovascular events in hemodialysis group aligns with contemporary literature that it is accounted for by thrombotic events, vein injury, fibrosis and stenosis associated with central vein access devices such as dialysis catheters [40–42]. The statistically insignificant differences in risk of all-cause infections between the 2 groups could be accounted for by the fact that both modalities are associated with dialysis devices induced infections [43–47]; peritoneal dialysis with peritonitis [48, 49] and hemodialysis with central vein access-devices infections [41, 42]. Statistical insignificant difference between the risk of lupus flares in the 2 comparison groups could be explained by the fact that SLE activity undergoes quiescence, “*burn out*”, when a lupus nephritis patient progresses to ESRD [15, 18] and during RRT as shown by *Gonzalez-Pulido* et al. (2014) [50], ideally due to immunosuppressants administration as illustrated by *Maroz* et al. (2013) [51]. A small study, by *Althaf* et al. (2014) [52] however, reported that the activity of SLE could exacerbate, preferably to lupus nephritis if the patient becomes pregnant. Regarding mortality, a study by *Mustapic* et al. (2013, 36), have reported more deaths to be associated with hemodialysis than peritoneal dialysis, specifically due to cardiovascular events.

According to our study, however, the mortality difference did not reach statistical significance. On the other hand, from our study, PD showed statistically significant benefits in terms of all-cause cardiovascular outcomes as compared to its counterpart group. This is supported by *Kang* et al. (2010) [36] who concluded the superiority of PD over HD. In another study, *Sekkarie* [53] shows no recovery advantage for patients treated by peritoneal dialysis as compared with hemodialysis, but in the same study, it was concluded that peritoneal dialysis preserves residual renal function better than hemodialysis.

The results of this study should be interpreted with caution. This is because of possible sources of biases observed at individual studies level as well as this review level. Other included studies used larger sample sizes while others used smaller sample sizes and none of the studies calculated sample sizes and power, thus introducing chances of type-1 error [54–56]. Participants had different mean age groups and some studies reported different comorbidities in their participants. Study settings were also different among included studies with 5 conducted in Taiwan, 3 in the United States of America, 2 in South Korea and 1 from China, Australia, Brazil, and the UK each. These different settings could be advantageous but could also mean different economical levels, different advancements in healthcare facilities. Furthermore, none of the studies was from Africa, thus reduced generalizability. A total of 2 studies, though fulfilling inclusion criteria were prospectively conducted while others were retrospectively conducted. Prospective studies could have lower chances of bias than retrospective studies [57, 58].

## Conclusion

Except for all-cause cardiovascular events in which PD is superior to hemodialysis offering better outcomes, both treatment modalities offer more or less similar clinical outcomes as effective initial choices of RRT in LN-ESRD patients prior to renal transplant. We, however, encourage further research on the question addressing better the possible sources of biases encountered in this study.

## Data Availability

The datasets used and analyzed during the current study are available from the corresponding author on reasonable request.

## Abbreviations

eGFR: Estimated glomerular filtration rate
ESRD: End-stage renal disease
LN-ESRD: Lupus nephritis end-stage renal disease
SLE: Systemic lupus erythematosus
SLEDAI: Systemic lupus erythematosus disease activity index
RRT: Renal replacement therapies
PRISMA: Preferred reporting items for systematic reviews and meta-analyses
KDIGO: Kidney disease- improving global outcomes
CKD: Chronic kidney disease
MeSH: Medical subject headings
HD: Hemodialysis
PD: Peritoneal dialysis
IR: Infection risk
LFR: Lupus flare risk
CVR: Cardiovascular events risk
MR: Mortality risk
PS: Patients survival
JS: Joel Swai (Author)
XZ: Xiexiong Zhao (Author)
JN: Julie-Raisa Noube (Author)
GM: Gui Ming (Author)
